# Analysis of chondroitin degradation by components of a *Bacteroides caccae* polysaccharide utilization locus

**DOI:** 10.1016/j.jbc.2025.110354

**Published:** 2025-06-07

**Authors:** Bernadette Alvarez, Olivia F. Canil, Kristin E. Low, Andrew G. Hettle, D. Wade Abbott, Alisdair B. Boraston

**Affiliations:** 1 Department of Biochemistry and Microbiology, University of Victoria, Victoria, British Columbia, Canada; 2 Lethbridge Research and Development Centre, Agriculture and Agri-Food Canada, Lethbridge, Alberta, Canada; 3 Department of Chemistry and Biochemistry, University of Lethbridge, Lethbridge, Alberta, Canada

**Keywords:** polysaccharide lyase, glycoside hydrolase, chondroitin, polysaccharide utilization locus, dehydratase, Bacteroides, microbiome

## Abstract

The human gut microbiota (HGM) possesses an enormously diverse capacity to metabolize both host and dietary glycans. Glycosaminoglycans (GAG) are complex polysaccharides that may be in the diet (*e*.*g*., from animal products) or may be presented by host tissues. These polysaccharides are known to be prioritized as a nutrient source by some members of the HGM. While significant advances in understanding how GAGs are metabolized by the HGM have been made, the varied architectures of the numerous polysaccharide utilization loci (PULs) targeting varied polysaccharides suggest that all the mechanisms of GAG degradation may not have been uncovered. Here we show that components of a (PUL) from *Bacteroides caccae* have activities consistent with comprising a unique pathway for depolymerization of chondroitin sulfate, a common GAG. After prior desulfation by an endo-sulfatase, BcSulf, to produce unsulfated chondroitin from chondroitin sulfate, the depolymerization pathway begins with the activity of a polysaccharide lyase from family 35, BcPL35. BcPL35 activity is coupled with BcGH88, an *exo*-**β**-uronyl hydrolase, and presumably BcGH109, a confirmed **α**/**β**-N-acetylgalactosaminidase. The most unique feature of the pathway is a **β**-D-glucuronate dehydratase, BcGDH, which we show through structural and functional analyses primes saturated non-reducing end **β**-D-glucuronate residues for hydrolysis by BcGH88. BcGDH is a member of a large family previously classified as glycoside hydrolase family 154. The potential reclassification of GH154 enzymes as uronate sugar dehydratases not only improves our understanding of chondroitin metabolism by *B*. *caccae* but will be broadly applicable to predicting the function of other pathways relevant to uronate sugar metabolism.

Bacteria of the phylum Bacteroidota (formerly Bacteroidetes) are well-established as glycan generalists, capable of degrading a complex array of substrates within the distal human gut (1, 2, 3, 4, 5). Members are known to arrange their carbohydrate metabolism-related genes in polysaccharide utilization loci (PULs), wherein genes are co-localized and co-regulated in response to a single glycan, or chemically related group of glycans (3, 6). In brief, PULs contain a complement of cell-surface glycan-binding proteins, CAZymes, carbohydrate sensors, and transcriptional regulators, as well as at least one pair of genes encoding SusC and SusD homologs. PULs are a major nutrient acquisition strategy for *Bacteroides* spp. (7, 8, 9, 10). Given the high prevalence of this genus in the HGM, the PUL system is intrinsically linked to the colonization of nutritional niches and the establishment of microbial ecosystems in the gut (8, 10).

*Bacteroides* spp. exhibit preferences for different glycans even when presented as a mixture in coculture growth experiments (7). Some prevalent species of this genus even prioritize glycosaminoglycans (GAGs) degradation over other glycans (6, 7). GAGs are ubiquitously distributed, linear heteropolysaccharides found in the extracellular matrix and on the cellular surfaces of mammalian cells (11, 12). The microbial degradation of GAGs generates biologically active microbial metabolites, such as short chain fatty-acids (SCFAs), that influence the host’s overall health state (4, 13) and modulate the microbial composition of the HGM (13, 14, 15). Members of *Bacteroides* genus can catabolize these recalcitrant glycan sources, allowing for the growth enrichment of other SCFA-producing gut microbes, which leads to increased levels of SCFAs that regulate colonic health and act as substrates for key metabolic processes (4, 5, 16, 17, 18). Thus, to appreciate how *Bacteroides* spp. interact with the host and other members of the HGM it is important to understand the molecular basis of how *Bacteroides* spp. interacts with GAGs as a nutrient source.

GAGs are separated into four main categories: hyaluronan (HA); heparin and heparan sulfate (HS); keratan sulfate (KS); and lastly, chondroitin sulfate (CS) and dermatan sulfate (DS) (11). Each group has a characteristic repeating disaccharide unit of a uronic acid (or galactose) paired with an amino sugar. GAG distinctions are based on the monosaccharide composition, sulfation level, and type of glycosidic linkage present. For example, chondroitin (CH) has a repeating disaccharide of glucuronic acid (GlcA) that is β-1,3-linked to N-acetylgalactosamine (GalNAc); the repeating disaccharide is β-1,4-linked. Chondroitin is typically sulfated to form CS. CS-A and CS-C are sulfated on the GalNAc residue at carbons four and 6, respectively. CS-D and CS-E comprise CS-C which is additionally sulfated at carbons two and 4, respectively of the GlcA (12).

*Bacteroides thetaiotamicron* is a widely used model organism to study microbe-host interactions, with extensively studied PULs that illustrate how it can target complex substrates, including GAGs (4, 5, 6, 19). However, less is known about how other *Bacteroides* spp. target these same substrates. *B*. *caccae* is a less studied member of the genus, with 60 predicted PULs in its genome (20, 21). Its abundance in the HGM is inextricably tied to diet, wherein it proliferates in high-fat and high-sugar diets (22). As with other members of its genus, *B*. *caccae* can degrade numerous glycan sources, including pectin (23), GAGs (4), and mucin (24). It is even capable of subsisting entirely on GAGs, including CS-A and CS-C, as its sole carbon source (6), and can enrich the growth of other *Bacteroides* spp. by sharing glycan degradation products (4).

The content of the PUL in the *B*. *caccae* (strain ATCC 43185) genome that is annotated as “PUL25” is unusual in its gene content. The predicted functions of the gene products include peptidases and CAZymes, including predicted members of PL35, GH88, GH109, and GH154 (Fig. 1*A*). None of these proteins have been functionally characterized. Other members of PL35 show lyase activity on the GAGs HA, HS, and CS (25, 26), while members of GH88, GH109, and GH154 families are reported to have unsaturated uronyl hydrolase (27, 28, 29, 30), hexosaminidase (31), and β-glucuronidase (9) activities, respectively. Hence, we hypothesize that the CAZymes encoded by PUL25 target a glycosaminoglycan. Through structure-function analyses of these enzymes, we show that the target substrate is most likely CH (*i*.*e*., chondroitin lacking sulfation). Unexpectedly, we found the predicted GH154 to lack glycoside hydrolase activity but rather it has a carbohydrate dehydratase activity that primes saturated non-reducing end β-glucuronic acid residues for cleavage by BcGH88, the unsaturated uronyl hydrolase. We also show that the founding member of the GH154 family, BT_3677 (32), also has dehydratase activity, leading us to propose that the entire family has this activity and is not a glycoside hydrolase family. Together, these results give new insight into GAG metabolism by bacteria, particularly members of the gut microbiota, and potentially reclassify GH154 as a novel family of dehydratases.

**Figure 1:**
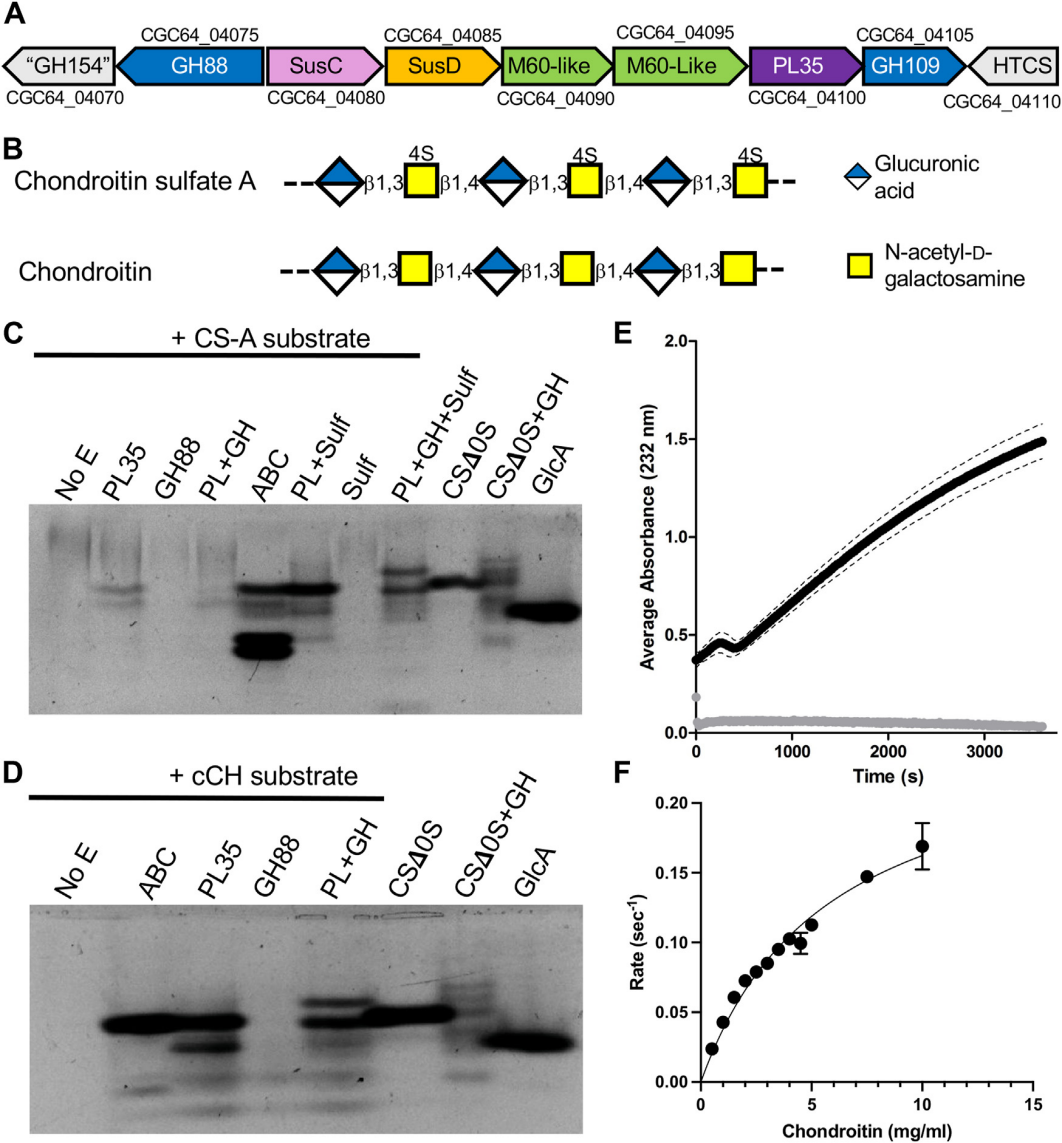
**Structure of BcPUL25 and activity of the keystone enzyme**. *A*, schematic of *B*. *caccae* PUL25 with initial annotations (*centered*) and locus tags (above and below).*B*, Schematics of chondoritin sulfate and chondroitin. *C*, activity analysis by FACE of BcPL35 (PL35 or PL), BcGH88 (GH88 or GH), BcSulf (Sulf), and combinations thereof on chondroitin sulfate, CS-A. ABC lyase is included as a positive control. CSΔ0S, CSΔ0S with BcGH88, and GlcA are included for reference. *D*, as in panel but without sulfatase and using chemically desulfated CS-A, cCH, as a substrate. *E*, progress curve of cCH (*black*) and CS-A (*grey*) cleavage by BcPL35. Dashed lines represent the standard deviation of triplicate samples. *F*, kinetic analysis of cCH cleavage by BcPL35 (see also Fig. S3). *Error bars* indicate the standard deviation of triplicate samples.

## Results

### BcPL35 from PUL25 prefers chondroitin

The founding member of the PL35 family was originally characterized to have *endo*-chondroitinase activity (*i*.*e*. on unsulfated chondroitin) (33), though additional members have been recently identified with broad GAG-lyase activity (25, 26). We screened recombinant BcPL35 against CS-A, CS-B (also known as dermatan), CS-C, and HA as possible substrates. Activity was only detected on HA in this initial screen (Fig. S1, *A*–*C*). Given the chondroitinase activity in the family, we sought to test the activity of BcPL35 on this substrate. BT_3349 from *B*. *thetaiotamicron* is a previously characterized chondroitin active *endo* 4-O-sulfatase (34). Its *B*. *caccae* ortholog is CGC64_04290 (BcSulf) from CAZyme cluster 1. As BcSulf has 92% amino acid sequence identity with BT_3349 and 100% conservation of the active site, we used recombinant BcSulf to determine if this putative chondroitin active *endo* 4-O-sulfatase would potentiate the activity of BcPL35 on CS-A. BcSulf was active on *p*NP-SO_4_, confirming its sulfatase activity. Treatment of CS-A with BcSulf rendered this otherwise recalcitrant polysaccharide a substrate for BcPL35 (Fig. 1*B* and S1*D*). The product produced by BcPL35 on BcSulf-treated CS-A had similar mobility by fluorophore-assisted carbohydrate electrophoresis (FACE) as an unsaturated, unsulfated chondroitin disaccharide standard (CSΔ0S) and with one of the three main products of CS-A digestion by *Proteus vulgaris* ABC lyase, which is known to produce primarily unsaturated disaccharide products (35). With this result in hand, we also used chemically desulfated CS-A (denoted as cCH) prepared using a previously described method (36). Again, the product produced by BcPL53 had similar mobility by FACE as CSΔ0S and the main product of CS-A digestion by ABC lyase (Figs. 1*C* and S2*B*).

The cleavage of cCH was also monitored by UV absorption at 232 nm to follow the generation of the Δ4,5-unsaturated bond typical of polysaccharide lyase activity (Fig. 1*D*). Consistent with the FACE results, BcPL35 displayed a time-dependent increase in product formation when using cCH as a substrate but not CS-A. A kinetic analysis using absorbance to quantify product formation allowed us to determine parameters of 15.6 (±1.8) min^-1^ for k_cat_ and 5.8 (±1.1) mg ml^-1^ for K_m_ (errors represent 95% CI). k_cat_/K_m_ was calculated to be 2.7 ml mg^-1^ min^-1^ (Figs. 1*E* and S3*A*). However, due to solubility issues with the substrate, we were unable to assay saturating substrate concentrations, therefore, we corroborated the k_cat_/K_m_ by three independent determinations of k_cat_/K_m_ at low concentrations of cCH (<one-fifth K_M_), which gave an average value of 2.8 (±0.5) ml mg^-1^ min^-1^ (error indicates the standard deviation of the independent replicates).

The FACE results suggested that the BcPL35 product was most likely predominantly CSΔ0S. To confirm this, we used LC-ESI-MS to analyze the products of cCH digestion by BcPL35. This confirmed that the major product from this reaction was CSΔ0S along with relatively lower amounts of unsaturated and unsulfated tetra- and hexasaccharide (Fig. S4 and Table S1). Sulfated oligosaccharide species were not readily detectable, consistent with either complete chemical desulfation of the substrate and/or strict specificity of the enzyme for non-sulfated stretches of polysaccharide.

### Structure and mutagenesis of BcPL35

**Figure 2:**
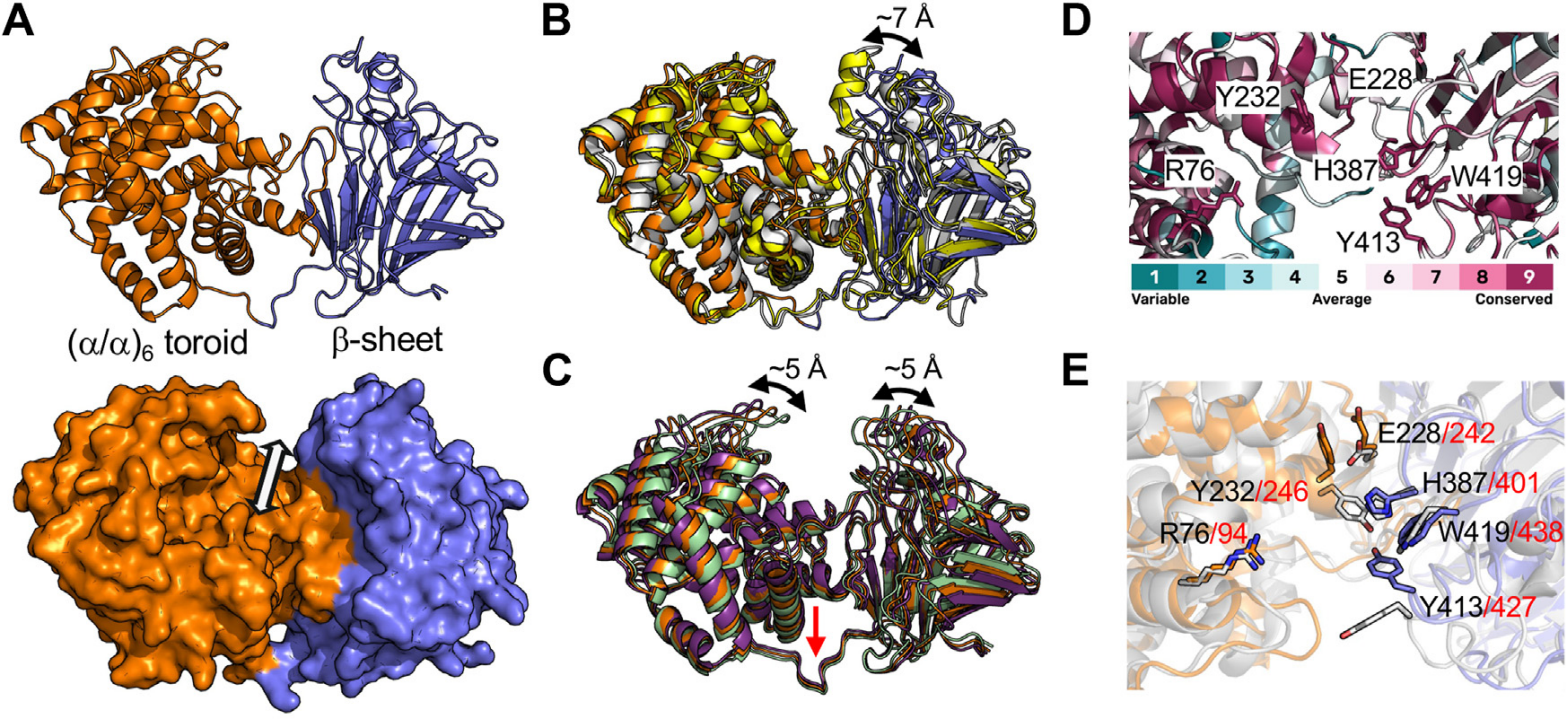
**Structural analysis of BcPL35**. *A*, cartoon and surface representations of the 1.75 Å resolution structure of BcPL35. The two domains are colored *orange* and *blue*. *B*, overlap of BcPL35 (*orange* and *blue*) with GAGase II (*grey*, PDB ID 8KHV) and GAGaseVII (*yellow*, PDB ID 8KHW). *C*, Cartoon representation of BcPL35 conformations determined by normal mode analysis with El Nemo compared with the crystal structure (*orange*). The predicted conformational extremes are shown in *green* and *purple* with the hinge point indicated by a *red arrow*. *D*, Consurf analysis of BcPL35. Reside conservation is color ramped teal to *purple* based on 150 randomly sampled homologs out of ∼1700 found with amino acid sequence identities between 25%-95%. Residues chosen for mutagenesis are shown as sticks. *E*, conservation of mutated residues with GAGase II (*grey*, PDB ID 8KHV).

BcPL35 crystallized in the spacegroup P2_1_2_1_2_1_ and we solved its structure to a resolution of 1.75 Å . The overall fold is a bi-domain structure of a (α/α)_6_ toroid fused to an anti-parallel β-sheet domain (Fig. 2*A*), thus generally similar to that of other PL families 8, 12, 15, 17, 21, 23, and 39 (37). The interface of the two domains is characterized by a deep groove that contains the putative catalytic machinery (Fig. 2*A*). The groove architecture of the active site that spans the full protein suggests endo-recognition of the substrate, which is consistent with the production of oligos with a degree of polymerization from two to six. The closest structural homologs of BcPL35 are GAGase II and GAGase VII, which are endo-acting PL35 enzymes from *Spirosoma fluviale* and *Bacteroides intestinalis* DSM 17393, respectively (38). The structures of both GAGase II (PDB ID 8KHV) and GAGaseVII (PDB ID 8KHW) have a root mean square deviation (RMSD) of 2.2 Å with BcPL35. The relatively high RMSD values appear to reflect that BcPL35 adopts a conformation with more widely separated N- and C-terminal domains (Fig. 2*B*). If the domains are separately overlapped with the respective domains in either GAGase II or VI the RMSD values drop to ∼1 Å, more accurately reflecting the high structural identity of the proteins. This did suggest, however, that there may be flexibility between the domains of BcPL35, which we interrogated by normal mode analysis using El Nemo (39). The primary predicted mode of flexibility involved a hinge motion at the separation between the domains resulting in ∼10 Å of movement between the domains with the crystal structure approximating an intermediate conformation (Fig. 2*C*). The most closed predicted conformation showed an RMSD of 1.8 Å with both GAGase II and GAGaseVII, respectively.

An analysis of conserved residues in the putative BcPL35 active site cleft allowed us to select six highly conserved residues for mutation to alanine: R76, E228, Y232, H387, Y413, and W419 (Fig. 2*D*). This includes the conserved proposed catalytic tyrosine and histidine in GAGase II (Y246, H401) (Fig. 2*D*) and GAGaseVII (Y241, H397), which are Y232 and H387 in BcPL35. Using cCH as a substrate, the activity for the R76 A, E228 A, H387 A, and Y413 A mutants was below detectable levels. The Y232 A mutant showed a roughly 3-fold reduction in activity relative to the wild-type with a k_cat_/K_m_ of 0.8 ± 0.1 ml mg^-1^ min^-1^ (Fig. S3*A*). The W419 A mutant had only slightly reduced activity of ∼60% wild-type with a k_cat_/K_m_ of 1.7 ± 0.1 ml mg^-1^ min^-1^ (Fig. S3*A*). This largely agrees with the dramatically reduced or absent activity of analogous mutants of GAGase II (38). The exception was W419 in BcPL35, which had minimal impact on activity whereas the corresponding W419 A mutation in GAGase II eliminated its activity. This may reflect the use of HA as a substrate for the GAGase II assays vs cCH in our BcPL35 assays and potential differences in the modes of substrate recognition of these polysaccharides.

### BcGH88 works in tandem with BcPL35

**Figure 3:**
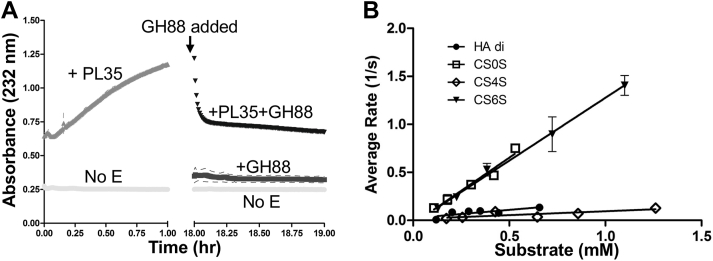
**Activity of BcGH88**. *A*, BcGH88 works on the products of BcPL35. cCH was treated with BcPL35 until equilibrium was reached, as judged by a plateau in absorbance. The reaction was then spiked with BcGH88. A no enzyme control was included; this was also spiked with BcGH88 at the same time. *B*, kinetic analysis of BcGH88 on different substrates, as indicated (see also Fig. S5*C*). *Error bars* indicate the standard deviation of triplicate samples.

The *exo-*uronyl hydrolase activity of the GH88 family targets Δ4,5-unsaturated glucuronyl or galacturonyl residues and through their unusual catalytic mechanism releases the saturated 5-keto-4-deoxyuronate product (40). These enzymes typically act after polysaccharide lyase activity to hydrolyze the newly formed unsaturated monosaccharide at the non-reducing end (28, 30, 41). We therefore tested BcGH88 on the products generated by BcPL35 substrate cleavage. Reactions of CS-A with BcPL35 and BcSulf, or cCH with just BcPL35 were supplemented with BcGH88 and analyzed by FACE. In both cases, the major products formed by BcPL35 were completely converted to two products by BcGH88, indicating the activity of BcGH88 on products produced by BcPL35 (Fig. 1, *B* and *C*). We further tested this using absorbance at 232 nm to track the formation of BcPL35 products followed by the addition of BcGH88 and the loss of absorbance owing to the removal of the terminal Δ4,5-unsaturated glucuronyl sugar and conversion to a non-absorbing species. As anticipated, after BcPL35 reactions were allowed to come to equilibrium, the addition of BcGH88 resulted in the rapid depletion of absorbance at 232 nm (Fig. 3*A*). We further pursued this by directly treating the disaccharide CSΔ0S, which has the non-reducing end Δ4,5-unsaturated glucuronyl residue, with BcGH88 and monitoring the reaction progress by loss of absorbance, indicating conversion of the Δ4,5-unsaturated glucuronyl residue (Fig. S5*A*). To provide additional evidence that BcGH88 produces the expected 5-keto-4-deoxyuronate product, we used a coupled assay utilizing KduI (5-keto-4-deoxyuronate isomerase) and KduU (2-dehydro-3-deoxy-D-gluconate dehydratase) (42). Qualitatively, the oxidation of NADH observed when CSΔ0S is cleaved by BcGH88 in the presence of KduI and KduU supports the production of the expected 5-keto-4-deoxyuronate product by BcGH88 (Fig. S5*B*). Notably, 5-keto-4-deoxyuronate has a free aldehyde and keto group, both of which can be labeled by the reductive amination used to introduce the AMAC fluorophore for FACE. This may explain the multiple labeled species observed by FACE when BcGH88 releases 5-keto-4-deoxyuronate (Fig. 1, *B* and *C*).

Using the assay for loss of absorbance at 232 nm, we assessed the kinetics of BcGH88 activity on a variety of Δ4,5-unsaturated disaccharides (Figs. 3*B* and S5*C*). The activity on Δ4,5-unsaturated heparin disaccharide (no sulfation) was too low to quantify. BcGH88 had activity on CSΔ0S and CSΔ6S with k_cat_/K_m_ values of 1.38 ± 0.11 s^-1^ mM^-1^ and 1.31 ± 0.11 s^-1^ mM^-1^, respectively, and therefore had no significant preference for either. The enzyme had roughly 10-fold lower efficiency on ΔHA and CSΔ4S with k_cat_/K_m_ values of 0.17 ± 0.04 s^-^1 mM^-1^ and 0.08 ± 0.02 s^-^1 mM^-1^, respectively.

### BcGH109 is an **α/β**-N-acetylgalactosaminidase

**Figure 4:**
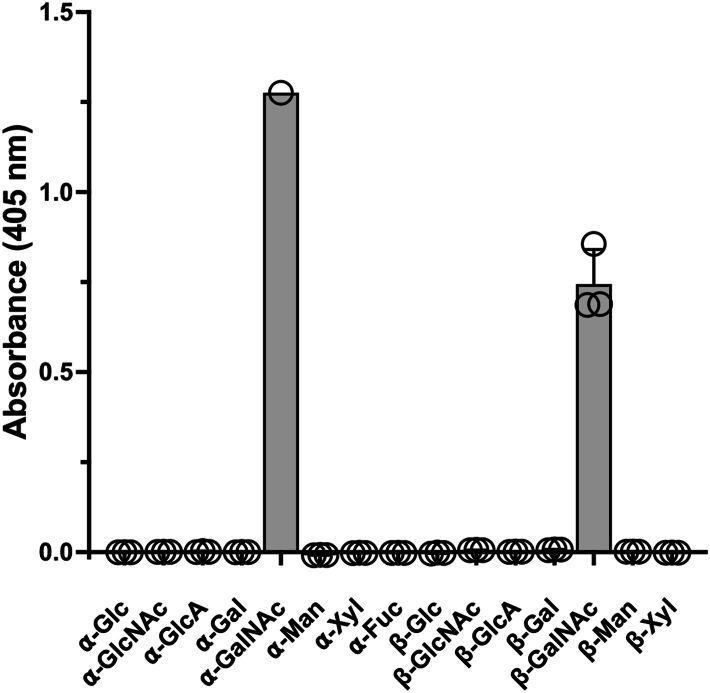
**Activity of BcGH109**. Hydrolysis of 4-nitrophenyl glycosides by GH109. Symbols indicate independent measurement and the bars indicate the average of these.

Recombinant BcGH109 was screened against a panel of aryl-glycosides to identify its substrate repertoire. Significant activity was only observed on *p*NP-α-GalNAc and *p*NP- β-GalNAc (Fig. 4), which is consistent with all other prior examples of the family being classified as N-acetylgalactosaminidases, often with activity on both α- and β-N-acetylgalactosamine moieties (31, 43, 44, 45). This suggested that the role of BcGH109 may be to cleave the terminal β-1,3-linked GalNAc left after BcGH88 activity on tetra- or hexasaccharide products of BcPL35. We attempted to examine this using FACE to separate reactions of CS-A with BcPL35, BcSulf, and BcGH88, or cCH with BcPL35 and BcGH88, which were supplemented with BcGH109. However, this was inconclusive, likely owing to the relatively small amounts of longer oligosaccharides produced by BcPL35.

### Identification of a novel dehydratase

To identify the activity of a recombinant version of the putative GH154 protein, we also screened it against a panel of aryl-glycosides on the premise it would have β-glucuronidase or β-galactosidase activity like other reported members of the family (9, 32). IMAC purified preparations of the protein produced from *Escherichia coli* BL21 did display glycoside hydrolase activity on some aryl-glycosides, most notably pNP-β-D-galactopyranoside (Fig. S6*A*). However, the activities were inconsistent between multiple individual preparations, leading us to believe that the activity was from contaminating proteins, such as LacZ. We, therefore, used a purification protocol that used the *E*. *coli* Tuner strain, in which LacZ is absent, and an additional size exclusion chromatography purification step after IMAC. We were unable to find any activity of these preparations on aryl-glycosides (Fig. S6*A*). Glycoside hydrolases occasionally have strict specificity for the sugar residue preceding the hydrolyzed glycosidic bond (*i*.*e*., in the −1 subsite or so-called “aglycon” specificity). We then used a linked assay to detect glucuronic acid released from chondrosine (GlcAβ-1,3-GalN), a saturated chondroitin disaccharide mimic, after treatment with the enzyme but failed to detect the monosaccharide (Fig. S6*B*).

**Figure 5:**
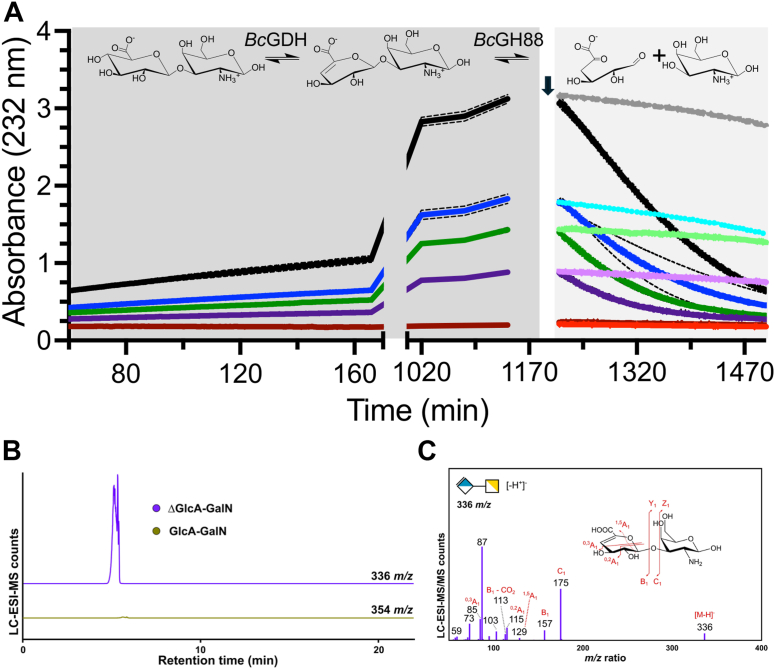
**The activity of BcGDH (GH154) on chondrosine**. *A*, BcGDH is active on chondrosine and BcGH88 is active on the products. Chondrosine at 0 mM (*dark red*), 0.1 mM (*dark purple*), 0.5 mM (*dark green*), 1.5 mM (*dark blue*), and 3.0 mM (*black*) was treated with BcGDH until equilibrium was reached, as judged by a plateau in absorbance. Reactions were in triplicate and *dashed lines* indicate the standard deviation of these readings. Two of each triplicate was then spiked with BcGH88 (indicated by an *arrow*), this data is shown in the corresponding *dark color*. Buffer was added to one of the triplicate samples, this data is shown in a light version of the corresponding color. The proposed reactions and structures are shown above. *B*, enzyme reaction products of chondrosine with BcGDH were analyzed by LC-ESI-MS with extracted ion chromatograms shown for ions of interest with ion counts scaled relative to the most intense peak. *C*, ESI-MS/MS with HCD was performed to identify the extracted ion for the primary catalytic product in each.

Seeking an alternative hypothesis for the activity of the putative GH154 protein, we noted that analysis of the interaction network of the GH154 family by STRING (46) revealed frequent co-occurrence and co-localization of GH154 encoding genes with genes encoding unsaturated uronyl hydrolases in the GH88 and GH105 families. A prior study revealed the role of a unique dehydratase, P29_PDnc, in generating a new Δ4,5-unsaturated non-reducing end, thus creating a substrate for an accompanying GH105 in ulvan depolymerization (47). Though GH154 is not related at the primary structure level to P29_PDnc, we hypothesized that our protein may be a dehydratase with a similar activity relationship to unsaturated uronyl hydrolases. We tested the activity of our protein on chondrosine by monitoring the absorbance at 232 nm to detect the formation of the predicted Δ4,5 double bond. The absorbance increased in a time-dependent manner, with the rate of increase dependent upon the chondrosine concentration (Fig. 5*A*). The efficiency of BcGDH-catalyzed generation of the absorbing species was determined to have a k_cat_/K_m_ of 0.54 (±0.02) min^-1^ mM^-1^ for chondrosine (Fig. S7, *A* and *B*). Reactions of BcGDH with chondrosine were allowed to go to completion and then spiked with either buffer or BcGH88. Only reactions spiked with BcGH88 showed a rapid decrease in absorbance, consistent with cleavage of the glycosidic bond by BcGH88 and indicating the formation of a Δ4,5-unsaturated glucuronyl residue on chondrosine (Fig. 5*A*). To confirm this, we used LC-ESI-MS to examine the structure of the reaction product. The mass of the reaction product, 336 Da (Fig. 5*C* and Table S1), was consistent with the formation of the dehydrated product while MS/MS analysis supported the structural assignment of the product as containing the Δ4,5 bond of dehydrated chondrosine (Fig. 5*D*). This protein, which was previously classified as a GH154, shows properties most consistent with dehydratase activity. Thus, we have renamed it BcGDH (GDH for glucuronic acid dehydratase).

On the basis of this result, we also tested the possible dehydratase activity of BT_3677, the founding member of GH154, using the absorbance assay to detect product formation. BT_3677 did not display activity on chondrosine but had activity on 1-*O*-methyl-β-D-glucuronate with a k_cat_/K_m_ of 0.35 (±0.01) min^-1^ mM^-1^ (Fig. S7, *A* and *B*). BcGDH did not have activity on 1-*O*-methyl-β-D-glucuronate, suggesting this family of proteins may have different specificity for the monosaccharide and/or linkage preceding the terminal glucuronic acid residue.

### Structural analysis of BcGDH

**Figure 6:**
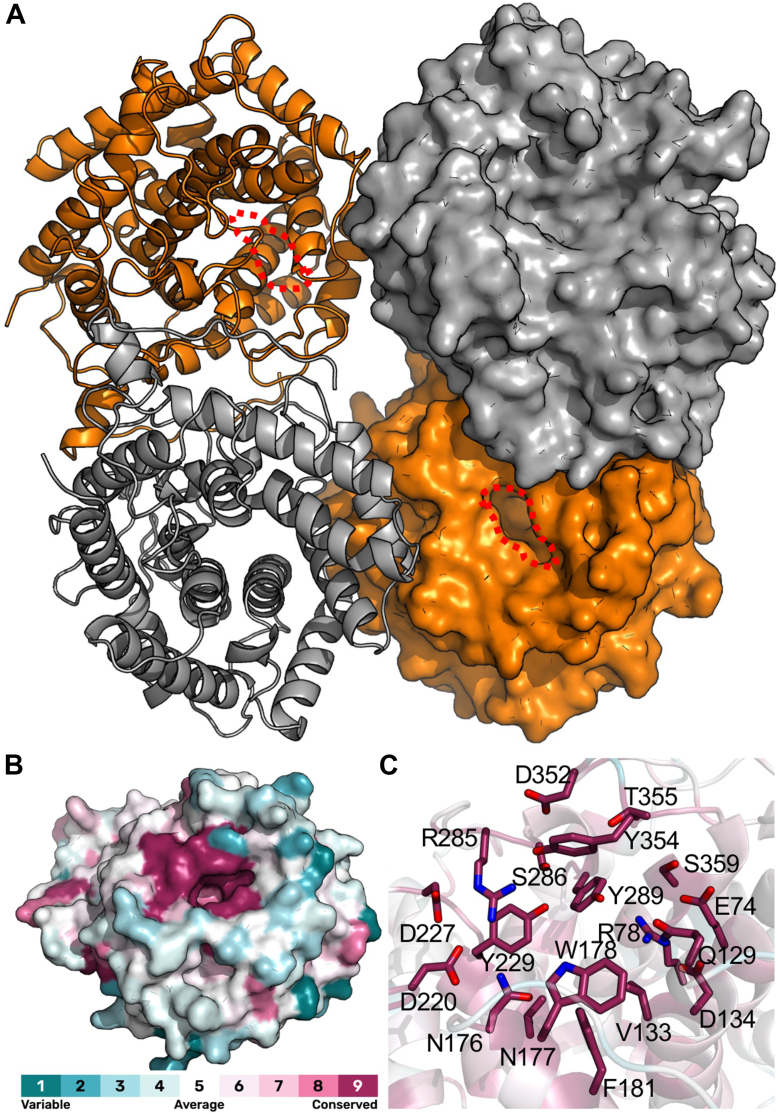
**Structural analysis of BcGDH**. *A*, structure of the BcGDH tetramer determined at 1.75 Å resolution. Two of the monomers are shown as cartoons while the other two are shown in surface representation. The active site is indicated with a *red dashed line*. *B*, Surface representation of a BcGDH monomer colored by Consurf analysis. Residue conservation is color ramped teal to *purple* based on 150 randomly sampled homologs out of ∼1800 found with amino acid sequence identities between 35%-95%. *C*, conservation in the active site, colored as in panel B, with conserved residues shown as sticks.

We solved the X-ray crystal structure of BcGDH and refined it in the spacegroup P2_1_ to 2.25 Å resolution. The resulting structure comprised a well-organized tetramer in the asymmetric unit that is predicted by PISA analysis to be stable in solution (Fig. 6*A*). We also obtained slightly lower resolution and lower quality diffraction data sets of BcGDH in P1, two different P2_1_2_1_2_1_ crystal forms, and a second P2_1_ crystal form. We chose not to refine these but noted that the same tetramer was present in all acquired crystal forms, supporting this as a stable quaternary structure.

The structure shows a pronounced slot on the surface of each monomer that is slightly tilted toward the center of the tetramer (Fig. 6*A*, circled region). An analysis of the surface residues of BcGDH for conservation in the family revealed the residues in the slot to be highly conserved and likely to comprise the active site (Fig. 6, *B* and *C*). Residues on the surfaces making up the quaternary structure interfaces were not conserved, possibly indicating different, or lacking, quaternary structures among other family members. Based on this analysis, we chose to make alanine mutations of 10 residues in the putative active site (E74 A, R78 A, N176 A, N177 A, W178 A, F181 A, Y229 A, R285 A, Y289 A, and Y354 A) and assay them for activity. None of the mutants had activity when using up to 10 mM chondrosine, despite displaying thermal stability similar to the unmutated enzyme (Table S2). We note, however, that chondrosine is a relatively poor substrate, likely by virtue of the substitution of what should be an acetamido group on N-acetylgalactosamine for the amino group on galactosamine. Any small perturbations in the enzyme–substrate interaction that even subtly decrease activity may render it below levels of reliable detection.

**Figure 7:**
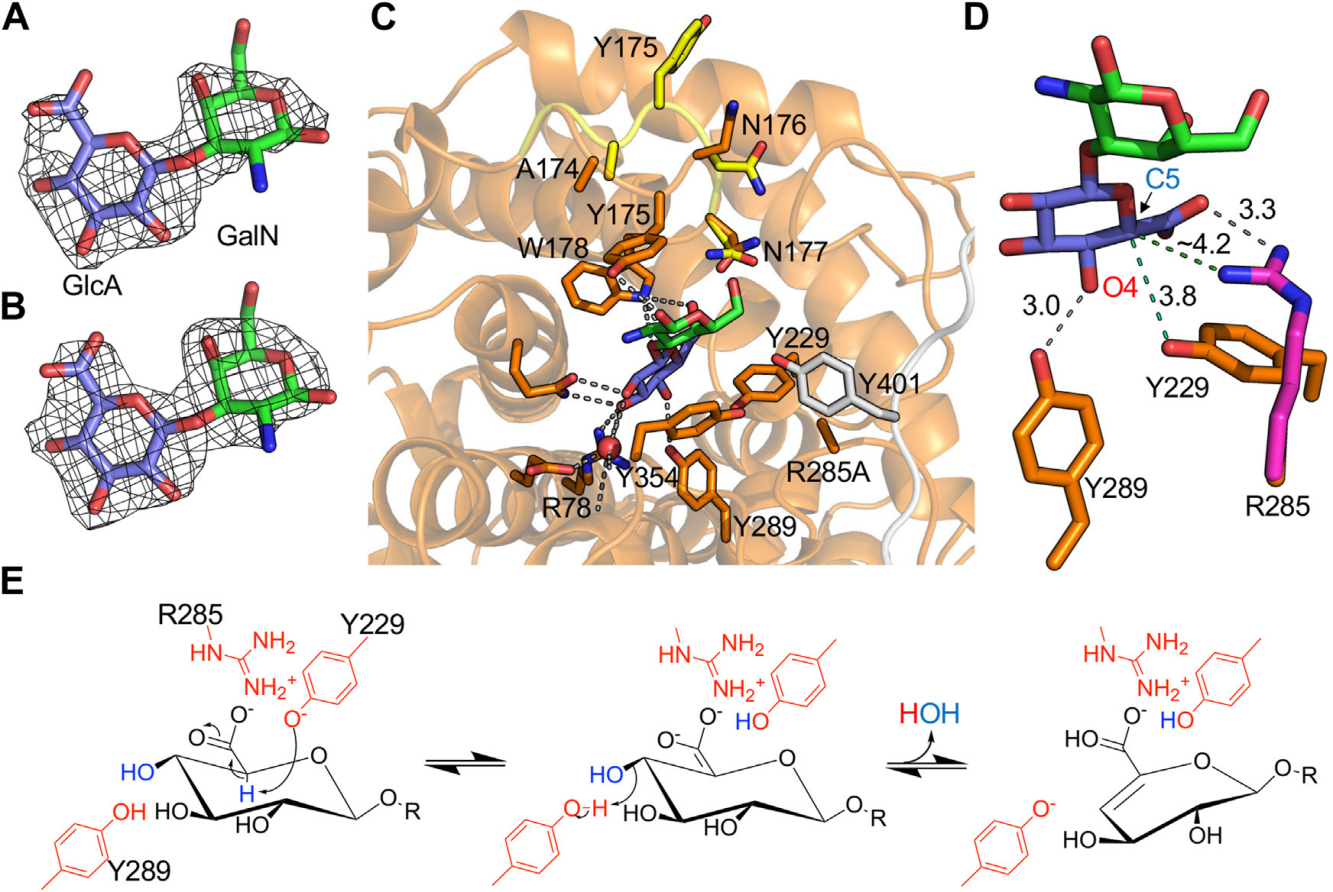
**Structure of BcGDH R285 A mutant in complex with chondrosine**. *A*, difference electron density map (F_o_-F_c_) contoured at 2.6σ determined by refinement in the absence of the ligand coordinates. *B*, electron density map (2F_o_-F_c_) contoured at 0.9σ determined by refinement with the ligand coordinates. *C*, interactions within the active site. The mobile loop is shown in both conformations with the *yellow colored loop* indicating the disengaged conformation. The *grey loop* containing Y401 is donated by a neighboring monomer in the tetramer. *D*, proposed key catalytic residues and distances in the active site. R285 (*magenta*) was placed *via* overlap with the unliganded structure. *E*, proposed catalytic mechanism.

To trap a substrate complex, we crystallized the inactive R285 A mutant and soaked crystals in chondrosine. The structure comprising a BcGDH tetramer in the asymmetric unit was refined in the spacegroup P2_1_2_1_2_1_ to 2.6 Å resolution. Electron density in the active site was clear and allowed modeling of the disaccharide (Fig. 7, *A* and *B*). A consortium of amino acid side chains in the active site, consistent with those predicted by conservation, were involved in interactions with the substrate (Fig. 7*C*). In this case, a tyrosine residue that is not conserved in the family is donated by a separate monomer in the tetramer and may help define the opening into the active site (Fig. 7*C*). The loop containing Y175 was modeled in two conformations. In one conformation the tyrosine sidechain is oriented out of the active site and makes interactions with another monomer in the tetramer (Fig. 7*C*). This is also the conformation observed in the unliganded structure. The other conformation is one where the sidechain of Y175 moves over the active site to interact with the galactosamine residue (Fig. 7*C*). The appearance of the loop in two conformations may reflect hindrance by the crystal packing to the structural change upon substrate binding or, possibly, incomplete occupation of the binding site by the substrate.

The structure of the trapped substrate on the inactive BcGDH R285 A mutant overlaid with the unliganded structure to estimate the position of R285 provides unique insight into the mechanism of this enzyme. Y289 is positioned in proximity to the O4 of the glucuronic acid and may act as a catalytic acid (Fig. 7*D*). The catalytic base is slightly more ambiguous as Y229 or R285 may perform the role of abstracting a proton from C5 of the glucuronic acid (Fig. 7*D*). However, Y229 is nearer the C5 proton and thus we propose it is more likely to perform the role. The role of R289, a mutant of which was completely inactive, may be two-fold. First, the proximity and positive charge on the guanidino group may assist with keeping the hydroxyl of Y229 in a deprotonated and negatively charged state for a role as a catalytic base. Second, there are no other obvious candidates to neutralize the negative charge on the glucuronic acid carboxylate and stabilize the oxyanion intermediate, but R285 is in sufficient proximity to this group to assist in this respect, though it is not in the typical geometry relative to the substrate carboxylate. Taken together, we proposed a two-step lyase catalytic mechanism of dehydration that by using two catalytic tyrosine sidechains resembles the alginate lyase mechanism proposed to be used by Alg17 C from *Saccharophagus degradans* (Fig. 7*E*) (48, 49).

## Discussion

GAGs are important molecules with profound biological impacts (5, 11). Except for HA, GAGs are generally found attached to a protein core, forming proteoglycans. In the human gut, altered abundance of specific proteoglycans and surface-bound GAGs play critical roles in inflammatory bowel disorders (4, 5). *Bacteroides* spp., including *B*. *caccae*, can break down numerous glycan sources (24), including GAGs, which it can use as a sole carbon source (6). Given the prevalence of the genus *Bacteroides* in the HGM (8, 10); its preference for GAGs as a nutrient source (6, 7); and its ability to generate SCFAs from GAGs (16, 18, 24); it is important to understand the molecular basis of how *Bacteroides* spp. targets GAGs for catalysis. Through biochemical analysis of the CAZymes from *Bacteroides caccae* ATCC 43185 (new assembly), we have uncovered support for an unusual biochemical cycle to depolymerize the GAG chondroitin sulfate.

The PL35 family was originally annotated as an *endo*-chondroitinase family, as its founding member from *Victivallis vadensis* (VvPL35) showed this activity (33). Like VvPL35, BcPL35 also displayed a preference for chondroitin, in this case produced by enzymatic (*i*.*e*., by BcSulf) or chemical desulfation. BcPL35 was also active on HA, which lacks sulfation and only differs from chondroitin by the substitution of GalNAc by GlcNAc. However, this appeared to make a relatively poor substrate, as judged by attempts to monitor reaction progress by UV absorbance, while the kinetic parameters on chemically desulfated chondroitin were similar to those observed recently for other PL35 enzymes on their preferred substrates (26). Consistent with other members of the GH88 family, BcGH88 displayed *exo-*uronyl hydrolase activity. Furthermore, the observation that BcGH88 had activity on the products of BcPL35 but poor activity on unsaturated disaccharides other than those derived from chondroitin, including the unsaturated disaccharide from HA, supports chondroitin as the main component of the substrate targeted by BcPUL25.

BcPL35 produces mainly unsaturated disaccharide, tetrasaccharide, and some hexasaccharide from chondroitin, which, *in vivo*, would most likely be produced by BcPL35 after enzymatic desulfation. However, it is possible the enzyme targets regions of chondroitin sulfate that are naturally low in sulfation. BcGH88 readily hydrolyzes the disaccharide product to the components 5-keto-4-deoxyuronate and GalNAc, which can feed into additional metabolic pathways (45, 50). The activity of BcGH88 on the tetrasaccharide would result in a GalNAcβ-1,3-GlcAβ-1,4-GalNAc trisaccharide. We postulate that the demonstrated β-N-acetylgalactosaminidase activity BcGH109 would translate to the removal of the terminal β-1,4-linked GalNAc, thus yielding the saturated chondroitin disaccharide repeating unit of GlcAβ-1,3-GalNAc. However, we were unable to confirm this due to the lack of substrate, or even substrate mimics.

**Figure 8:**
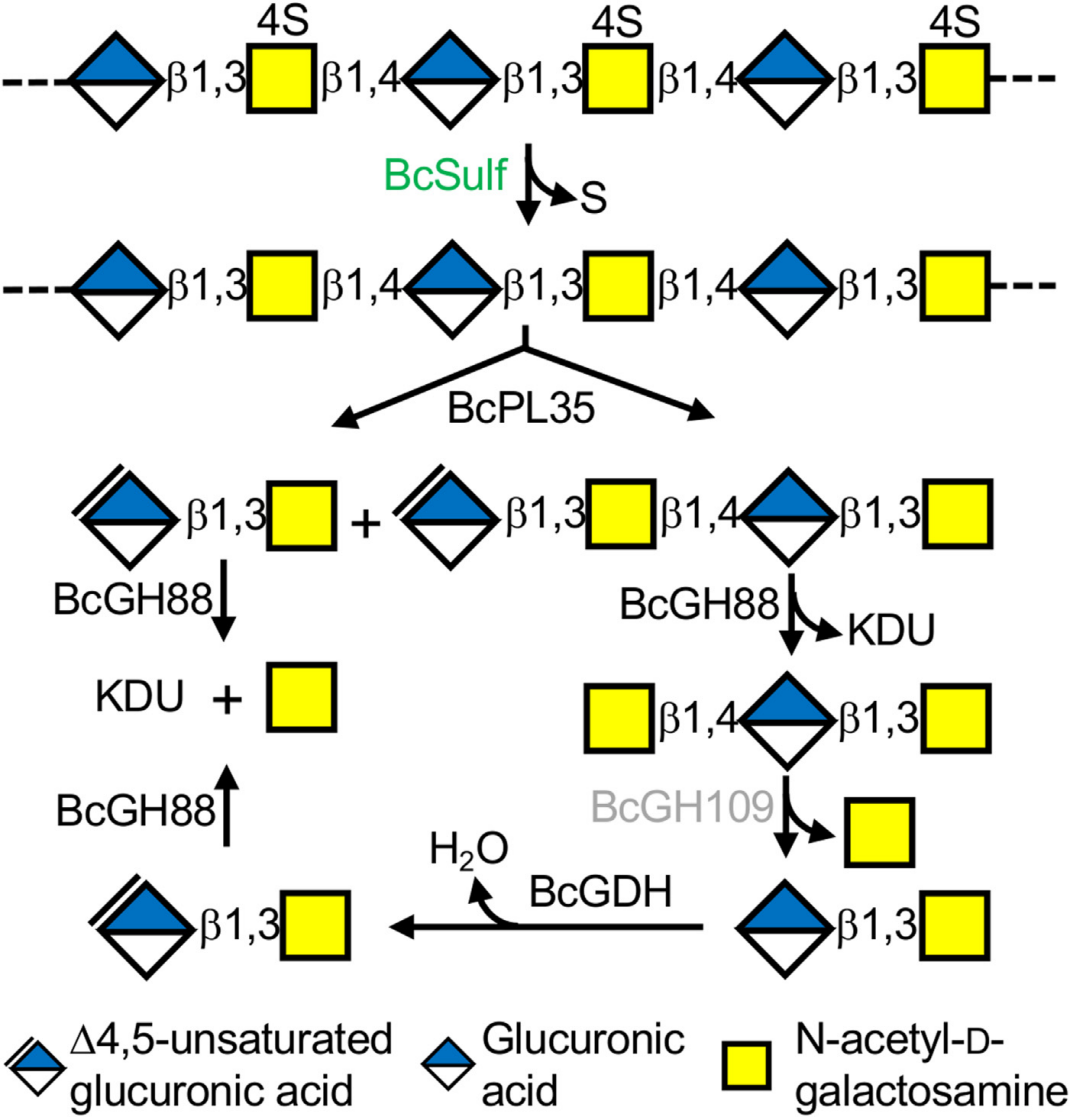
**Schematic of the chondroitin depolymerization pathway beginning with CS-A. Steps not encoded by BcPUL25 are shown in *green*.** BcGH109 has confirmed β-N-acetylgalactosaminindase activity but activity on chondroitin or a mimic has not been demonstrated so it is colored *grey*.

The first gene in BcPUL25 encodes a protein that, by its high sequence identity to members of glycoside hydrolase family 154, is annotated as a putative β-glucuronidase (9). This hypothesized activity was entirely consistent with the potential need to hydrolyze terminal saturated β-1,4-linked GlcA residues, as suggested would be revealed in the reactions above, but we were unable to find any glycoside hydrolase activity for the recombinant protein. Instead, the evidence using chondrosine as a mimic of the natural substrate clearly supports that this enzyme, which we refer to as BcGDH, is a β-D-glucuronic acid dehydratase that converts the terminal β-1,4-linked GlcA into a terminal Δ4,5-unsaturated uronic acid and, therefore, renders the sugar a substrate for BcGH88 (Fig. 5*A*). This would close the loop and allow the cyclical pathway as proposed to depolymerize the BcPL35 cleavage products of any length, or indeed any chondroitin fragment with either a non-reducing end GalNAc or GlcA, with the latter being either saturated or unsaturated (Fig. 8).

The two other characterized members of the GH154 family, BT3677 and BD-β-Gal, were reported to have *exo*-β-1,6-glucuronidase and *exo*-β-1,6-galactosidase activity, respectively (9, 32). BcGDH has 22% and 50% amino acid sequence identity with BT3677 and BD-β-Gal, respectively. The uncomplexed structure of BD-β-Gal has been determined to 1.76 Å resolution (PDB ID 8OI4). It displays the same tetrameric organization as BcGDH while monomers of each protein have a root mean square deviation of <0.8 Å (Fig. S8*A*). The residues in BcGHD that interact with the terminal GlcA are highly conserved across the family (Fig. 6, *B* and *C*) and essentially invariant with BD-β-Gal and BT3677, as judged by overlays with the crystal structure of BD-β-Gal and an AlphaFold three model of BT3677 (Fig. S8, *B* and *C*). None of the structures display an active site architecture consistent with typical glycoside hydrolase activity (*i*.*e*., potential acid/base or nucleophilic side chains in appropriate proximity to the substrate glycosidic bond). This strongly supports the contention that the “GH154” family is actually a family of carbohydrate dehydratases. This is additionally substantiated by the observation that BT3677 also displays dehydratase activity 1-*O*-methyl-β-D-glucuronate. The predicted structure of BT3677, however, reveals a second domain that contributes sidechains to the vicinity of the active site that is absent in BcGDH (Fig. S8*C*). Likewise, BD-β-Gal has a loop that contributes additional sidechains on the rim of the active site and is oriented into the catalytic pocket (Fig. S8*B*). Such accessorizing of the entrance to the active site may help dictate substrate selectivity. For example, BT3677 was active on 1-*O*-methyl-β-D-glucuronate but not active on chondrosine, while BcGDH showed a reversed pattern of selectivity.

The pathway of chondroitin degradation, therefore, utilizes an unusual cycle that pairs a dehydratase with an *exo*-uronyl hydrolase, in this case, a GH88, to remove a non-reducing end uronate monosaccharide (Fig. 8). This has only been observed before in ulvan degradation where P29_PDnc, a dehydratase that is unrelated to BcGDH at the amino acid sequence level, is paired with a GH105 *exo*-uronyl hydrolase (47). The protein family to which BcGDH belongs contains over 6000 members (37), suggesting this model of uronate sugar removal may be widespread. Indeed, genes encoding these dehydratases frequently co-occur and co-localize with genes encoding GH88 or GH105 *exo*-uronyl hydrolases. In the case of BcPUL25, the pairing of chondroitin-specific BcPL35 with BcGH88, BcGDH, and BcGH109 creates a logical cascade to completely depolymerize chondroitin and bypasses the need for a dedicated β-glucuronidase. BcPUL25, however, lacks a gene encoding a chondroitin sulfate-specific sulfatase while it somewhat cryptically contains genes encoding M60-like proteins, which are putative *O*-glycopeptidases (51). It is possible that BcPUL25 cooperates with the enzymes produced from other co-transcriptionally controlled genes, with the gene encoding BcSulf (CGC64_04290) being a logical candidate that would render at least some species of chondroitin sulfate (*e*.*g*., CS-A) a substrate for BcPUL25. However, the presence of putative *O*-glycopeptidases generally suggests a more complex target for BcPUL25. Chondroitin sulfate is typically a component of proteoglycans where the chondroitin sulfate is covalently attached to a protein backbone, such as in the lecticans, of which aggrecan from cartilaginous tissue is a prominent and abundant member (52, 53). In addition to chondroitin sulfate modifications, the protein backbone of aggrecan also bears mucin-like *O*-glycans (52), which may be the target of the putative *O*-glycopeptidases. Thus, the unique GAG degradation pathway encoded by BcPUL may be coupled with a greater capacity to target proteoglycans, again invoking the concept of the incredible metabolic diversity of the HGM.

## Experimental procedures

### Materials

Chondroitin sulfate A (CS-A), CS-B, CS-C, hyaluronic acid (HA), and all tested *p*NP-sugars were acquired from Sigma. All unsaturated disaccharides (ΔHA, ΔHep, CSΔ0S, CSΔ4S, and CSΔ6S), as well as chondrosine and methyl-β-D-glucuronide, were ordered from Biosynth. Saturated hyaluronic acid disaccharide (sHA) was obtained from Sussex Research.

### Protein production and purification

All constructs were obtained as synthetic genes in pET28a from Genscript or TwistBiosciences. Plasmids containing BcPL35 (CGC64_04100; residues 27–613), BcGH88 (CGC64_04075; residues 31–433), BcGH154 (CGC64_04070; BcGDH; residues 24–423), endo-sulfatases from *B*. *thetaiotamicron* (BT3349; BtSulf_S93 C; residues 1–508) (34), and its *B*. *caccae* ortholog (CGC64_04290; BcSulf_S91 C; residues 20–515), as well as GH154 from *B*. *thetaiotamicron* (BT_3677; residues 30–673) (32). BcPL35 and BcGDH mutant constructs were cloned using site-directed mutagenesis *via* polymerase chain reaction. All reading frames of the engineered genes incorporated an N-terminal 6-histidine tag separated from the target protein by a thrombin protease cleavage site. Oligonucleotide primers and DNA fragments used to generate mutants are given in Tables S3 and S4. The fidelity of all constructs was confirmed using bidirectional sequencing.

Proteins were produced in *E*. *coli* strain BL21 (DE3). KdgF, Kdul, and KduD were produced and purified as described previously (42). For assaying preliminary activity against *para*-nitrophenyl-containing (*p*NP) sugars, BcGH154 was expressed in *E*. *coli* strain Tuner (DE3) to account for the endogenous β-galactosidase in BL21 strains. In brief, cultures were grown at 37 °C, with shaking at 170 rpm, until an OD_600_ of ∼0.6 was reached. These growths were moved to 16 °C for at least 30 min prior to the addition of isopropyl β-D-1-thiogalactopyranoside (IPTG) (0.5 mM final concentration) to induce overnight expression of the target protein.

Mutant sulfatases, where the catalytic serine was mutated to cysteine to enable maturation in *E*. *coli*, were co-expressed with a sulfatase maturing enzyme (*Mycobacterium tuberculosis* formylglycine generating enzyme; MT_FGE) as described previously (54). Upon reaching an OD_600_ of ∼0.6, sulfatase cultures were moved to 16 °C prior to the addition of 250 mg L-arabinose to induce the production of MT_FGE. After 3 hours, IPTG was added to induce the production of target sulfatases. All proteins were left to express overnight. Maturation of sulfatases was confirmed *via* colorimetric assay with *p*NP-SO_4_, wherein increased absorbance at 405 nm indicated activity.

Target proteins were purified from clarified cell extracts *via* Ni^+2^-NTA immobilized metal affinity chromatography, wherein the target protein was eluted using a gradient of 20 to 500 mM imidazole in binding buffer (20 mM Tris pH 8.0, 500 mM NaCl). Fractions were assessed for purity using SDS-PAGE. Chosen fractions were pooled and dialyzed overnight at 4 °C. Isolated BcPL35 was either dialyzed into crystallography buffer (20 mM Tris pH 8.0, 500 mM NaCl, 10 mM dithiothreitol) or into activity buffer (20 mM Bis-Tris pH 6.0, 500 mM NaCl, 10% glycerol). Similarly, both BcGH88 and BcGH154 were dialyzed into a binding buffer for activity assays. For crystallography, BcGH154 was desalted into 20 mM Tris pH 8.0 (PD MidiTrap G-25; Cytiva) prior to overnight thrombin cleavage of the His-tag. KdgF, Kdul, and KduD were desalted into 20 mM Tris pH 7.4 buffer.

Additional purification of protein for crystallography was done using size exclusion chromatography (SEC) using a HiPrep 16/60 Sephacryl S-200 HR column (GE Healthcare) equilibrated in an appropriate buffer. All proteins were concentrated using a stirred ultrafiltration unit (Amicon) with a 10 kDa molecular weight cut-off membrane (EMD Millipore).

Protein concentration was determined using UV absorbances at 280 nm. Extinction coefficients were calculated using Expasy ProtParam. Concentrated proteins were stored at 4 °C, or flash frozen in liquid nitrogen and stored at −80 °C, prior to use.

### Generation of chondroitin

Chondroitin was made *via* chemical desulfation of chondroitin sulfate A (CS-A) using previously described methods (36). Briefly, CS-A-pyridine salt was prepared by dissolving 1.40 g of sodium CS-A in 40 ml dH_2_O. This was passed through a cation exchange column (Dowex 5W, X8, 50–100 mesh; Sigma). After washing the column with three column volumes of dH_2_O, the washes were pooled and neutralized to pH seven using pyridine prior to lyophilization. The final reaction used 0.53 g of CS-A-pyridine dissolved in 50 ml DMSO in 10% methanol and incubated at 80 °C for at least 5 h. This solution was diluted in an equivalent volume of dH_2_O, pH adjusted to between pH 9.0 to 9.5, and dialyzed in dH_2_O overnight, with multiple dH_2_O changes per hour the following day. Afterward, the chondroitin solution is filtered, lyophilized, and stored at −20 °C. The substrate was resuspended in dH_2_O prior to use.

### Fluorophore-assisted carbohydrate electrophoresis (FACE)

All enzymatic reactions for analysis by FACE were done at 37 °C for 2 hours, with 3 mg/ml GAG substrate, 5 μM per enzyme, 100 mM Tris pH 7.5, 1 mM MgCl_2_, and 10 μM sulfatase (when used). 2-aminoacridone (AMAC) was used to label reactions. Reaction progression was halted by adding 95% ethanol. Samples were dried in a speed vacuum for at least 2 hours, or until samples had a jelly-like consistency. These were resuspended in 0.02 M AMAC dissolved in diluted acetic acid (3:17, acetic acid: dH_2_O) and 0.1 M sodium cyanoborohydride in DMSO (NaBH_3_CN; Sigma) prior to overnight incubation at 37 °C, wrapped in foil. AMAC-tagged reactions were dried in a speed vacuum for at least 2 hours and resuspended in FACE loading buffer (62 mM Tris pH 6.8, 0.1% glycerol). All reactions were run on 30% polyacrylamide gels at 100V for 30 min, and 200V for 120 min after a 15-min rest time, in an icebox (4 °C) prior to imaging.

### UV absorption assays for activity

BcPL35 activity on various GAGs was initially assessed by absorbance at 232 nm following the generation of the Δ4,5 bond at the new non-reducing end. All reactions were done in triplicate in UV-Star 96-well microplates (Greiner Bio-One) and read at 232 nm for 1 h at 37 °C. Samples that displayed activity were left overnight at room temperature to reach equilibrium. BcGH88 was then added to these reactions and read under the same parameters as above. Reaction progression was monitored with a SpectraMax M5 plate reader (Molecular Devices). Final reactions comprised 100 mM Tris pH 7.4, 1 mg/ml chondroitin, and 1 μM per enzyme.

Kinetic analysis of chondroitin cleavage by BcPL35 at 37 °C was done through the continuous monitoring of double bond formation at 232 nm using substrate concentration up to 7 mg/ml. Initial assessment of all BcPL35 mutants was done with 1 μM enzyme, 3 mg/ml of chondroitin, and 100 mM Tris pH 7.5, in triplicate at 37 °C. Upon confirmation of activity, the kinetics of mutants with measurable activity were determined as wild type. Reactions were done in a UV-Star 96-well microplate (Greiner Bio-One) and read in a SpectraMax M5 plate reader (Molecular Devices). Saturation could not be reached, so k_cat_/K_m_ values were determined by linear fits to the plots of rate vs chondroitin concentration.

Cleavage of Δ4,5-unsaturated GAG-derived disaccharides by BcGH88 was monitored by loss of absorbance at 232 nm. All reactions were done in triplicate in UV-Star 96-well microplates (Greiner Bio-One) and incubated for 1 h at 37 °C. Reaction progression was monitored with a SpectraMax M5 plate reader (Molecular Devices). Reaction components include: 100 mM Tris pH 7.4, 0.500 mM GAG disaccharide, and 0.025 μM or 1 μM BcGH88. Kinetic assays were performed by continuous assay of A232 nm depletion at substrate concentrations up to 1.5 mM.

The activity of BcGH154 (aka BcGDH) on chondrosine was followed by increased absorbance at 232 nm. As with BcPL35, these reactions were allowed to plateau followed by the addition of BcGH88 and continued monitoring at 232 nm. All reactions were done in triplicate in UV-Star 96-well microplates (Greiner Bio-One) at 25 °C in a SpectraMax M5 plate reader (Molecular Devices). BcGDH and BcGH88 were used at 1 μM final concentration in 100 mM Tris pH 7.4. BcGDH mutants were qualitatively assessed for activity using 10 mM chondrosine in a continuous A232 nm assay. Kinetic assays were performed by continuous assay of A232 nm at substrate concentrations up to 6 mM. Kinetic analysis of BT3677 on 1-*O*-methyl-β-D-glucuronide was done under identical conditions using substrate up to 10 mM concentration.

In all kinetic assays, the absorbance was converted to concentration using the extinction coefficient of 5200 M^-1^ cm^-1^. Time windows of 10 min at the beginning of the kinetic runs were used to determine the initial rates. In all cases, saturation could not be reached, so k_cat_/K_m_ values were determined by linear fits to the plots of rate vs substrate concentration.

### Screening glycoside hydrolase activity

Initial screening with synthetic aryl-glycosides was done to explore the potential hydrolase activity of BcGDH and BcGH109. All reactions were done in triplicate using Costar 96-well microplates (Corning Incorporated) and incubated for 1 h at 37 °C. Reactions comprised 100 mM Tris pH 7.4, 1 mM aryl-glycoside, and 2 μM enzyme. Reaction progress was stopped by adding 100 μl of 100 mM NaOH, and endpoint results by absorbance at 405 nm were read with a SpectraMax M5 plate reader (Molecular Devices).

### Coupled assay for **β-**glucuronidase detection

β-glucuronidase activity on chondrosine was assessed using a coupled assay based for free glucuronic acid detection (Megazyme). The assay is based on oxidation of glucuronic acid by uronate dehydrogenase to generate NADH from NAD^+^. Reactions were performed in triplicate according to the manufacturer’s instructions in UV-Star 96-well microplates (Greiner Bio-One). Reaction progress was monitored at 37 °C for 30 min using a SpectraMax M5 plate reader (Molecular Devices), wherein reads were taken at 340 nm (55). Reaction progress was monitored at 37 °C for 30 min using a SpectraMax M5 plate reader (Molecular Devices), wherein reads were taken at 340 nm. Final reaction conditions include 50 mM Tris pH 7.4, 1.5 mg/ml chondrosine or glucuronic acid standard, and 5 μM BcGDH.

### Coupled assay for 5-keto-4-deoxyuronate detection

The activity of BcGH88 on unsaturated chondroitin disaccharide (CSΔ0S) was indirectly confirmed using a coupled assay with KduI and KduD for the detection of the BcGH88 product 5-keto-4-deoxyuronate. KduD activity consumes NADH, providing a readout at 340 nm. All reactions were done in triplicate in UV-Star 96-well microplates (Greiner Bio-One) and read at 340 nm for 1 h at 25 °C. Reactions included 100 mM Tris pH 7.4, 0.200 mM NADH, 0.500 mM CSΔ0S disaccharide, and 0.500 μM per enzyme. The substrate was equilibrated in a plate reader for 15 min prior to enzyme addition. Reaction progression was monitored with a SpectraMax M5 plate reader (Molecular Devices).

### Mass spectroscopy

Mass spectrometry was used to further clarify the final products of BcPL35 and BcGDH. Reactions were run overnight at 37 °C, flash frozen with liquid nitrogen, and stored at −80 °C prior to use. The final reactions were in 100 mM Tris pH 7.4 with 1 μM enzyme and 200 μg substrate. Chondroitin and chondrosine enzyme reaction products (100 μg) were run through a Supelclean ENVI-Carb SPE column (Sigma) whereby columns were preconditioned with 2 ml of 80% (v/v) acetonitrile 0.1% (v/v) TFA, and 6 ml of water. Reaction products were then added, washed with 10 ml water followed by 2 ml of 5% (v/v) acetonitrile 0.1% (v/v) TFA, and eluted using 8 ml of 80% (v/v) acetonitrile 0.1% (v/v) TFA and evaporated to dryness under nitrogen gas. Each sample was then resuspended in 150 μl of water and filtered (0.20 μm) before injection and separation. Liquid chromatography was performed on a Vanquish ultra-high performance liquid chromatography (UHPLC) system (Thermo Scientific). Separation of the chondroitin and chondrosine enzyme products was achieved using an Acquity UPLC BEH Amide (HILIC) Column, 130 Å, 1.7 μm, 2.1 mm × 150 mm (Waters) at a flow rate of 400 μl/min at 30 °C, using a gradient as shown in Table S5.

Enzyme products were injected in a volume of 10 μl for electrospray ionization mass spectrometry (ESI-MS) on an Orbitrap Fusion Tribrid system (Thermo Scientific) in negative ion mode. Mass spectra parameters are shown in Table S6. To select ions for MS2 experiments, a data-dependent MS2 strategy was employed using expected m/z values as listed in Table S1, and a dynamic exclusion filter was used after one time for 2.5 s with default mass tolerance values. Higher-energy collisional dissociation (HCD) was employed to generate fragments. MS spectra were analyzed using Xcalibur and Freestyle software packages (Thermo Scientific), and product ions were identified based on manual interpretation.

### Crystallography

Crystallization conditions for BcPL35 and BcGDH were initially screened using MCSG-1 to 4 (Anatrace) crystal screen kits and the sitting drop method of vapor diffusion in 96-well plates. Optimized BcPL35 native crystals were obtained at 18 °C *via* hanging drop vapor diffusion from protein concentrated to 8.6 g/L mixed at a 1:1 ratio with well solution containing 100 mM MES:NaOH pH 6.5, 300 mM MgCl_2_, and 12.5% PEG 4000. BcGDH crystals were obtained at 18 °C from protein concentrated to 26 g/L mixed at a 1:1 ratio with a crystallization solution containing 220 mM KCl and 24% PEG 3350. BcGDH R285 A at 18 g/L crystallized in 0.25 M sodium iodide, 5 to 13% glycerol, and 8 to 13% PEG 3350. The latter crystals were soaked for 30 min in a crystallization solution containing 50 mM chondrosine. All crystals were cryo-protected in crystallization solution supplemented with 20% ethylene glycol prior to mounting directly in a nitrogen cryo-stream at 100 K for data collection. The cryo-protecting solution for BcGDH R285 A also contained 40 mM chondrosine. X-ray diffraction data was collected in-house on a system comprising a Rigaku MM-007HF X-ray generator with a VariMaxTM-HF ArcSec Confocal Optical System and an Oxford Cryostream 800.

All datasets were processed using HKL2000. Structures were solved using AlphaFold2 predicted models for molecular replacement using PhaserMR. All models were manually corrected by building in COOT with refinement using REFMAC and/or phenix.refine. Waters were added in COOT with FINDWATERS and manually checked after refinement. In all datasets, refinement procedures were monitored by flagging 5% of all observations as “free”. Model validation was performed with MOLPROBITY. All data processing and model refinement statistics and PDB ID accession codes are shown in Table S7.

### Protein stability

The stability of BcGDH mutant proteins was assessed by differential scanning fluorimetry as reported previously (55). Protein was used at a concentration of 0.1 mg ml^-1^ in 20 mM Tris (pH 7.5) and 8 × SYPRO Orange (Invitrogen). Reaction mixtures were incubated in an Applied Biosystems 7500 Fast-Real Time PCR machine at 25 to 95 °C with a ramp rate of 1 °C min^-1^. Data was analyzed using the accompanying StepOne software (version 2.3).

## Data availability

The Protein Data Bank (PDB) accession codes for the structure coordinates and structure factors are 9O3Q (BcPL35), 9O4U (BcGDH), 9NWF (BcGDH R285 A chondrosine complex).

## Supporting information

This article contains supporting information.

## Conflict of interest

The authors declare they have no conflicts of interest with the contents of this article.
