# Supplementary material for: Analysis of chondroitin degradation by components of a Bacteroides caccae polysaccharide utilization locus
Source: J Biol Chem. 2025 Jun 7;301(7):110354. doi: 10.1016/j.jbc.2025.110354 (PMC12269599; doi:10.1016/j.jbc.2025.110354)
Supplement: Supplementary Material [file mmc1.docx]

Supplementary material for:

**Analysis of chondroitin degradation by components of a *Bacteroides caccae* polysaccharide utilization locus.**

Bernadette Alvarez^1^, Olivia Canil^1^, Kristin E. Low^2^, Andrew G. Hettle^1^, D. Wade Abbott^2,3^, and Alisdair B. Boraston^1^*.

^1^Department of Biochemistry and Microbiology, University of Victoria, PO Box 1700 STN CSC, Victoria, British Columbia, V8W 2Y2, Canada.

^2^ Lethbridge Research and Development Centre, Agriculture and Agri-Food Canada, Lethbridge, AB, Canada

^3^ Department of Chemistry and Biochemistry, University of Lethbridge, Lethbridge, AB, Canada

***Corresponding author:** Alisdair Boraston ([boraston@uvic.ca](mailto:boraston@uvic.ca)),

**This file includes:**

Tables S1 to S7

Figures S1 to S8

**Table S1:** Enzyme products as observed by LC-ESI-MS.

| **Species** | **Ion** | **Expected m/z** | **Observed m/z** | **Retention Time (peak) (min)** |
| --- | --- | --- | --- | --- |
| GlcA-GalNAc | [M-H]- | 396.1147 | 396.1124 | 5.13 |
| ∆GlcA-GalNAc | [M-H]- | 378.1042 | 378.1020 | 4.58 |
| GlcA-GalNAc-GlcA-GalNAc | [M-H]- | 775.2262 | 775.2227 | 6.88 |
| ∆GlcA-GalNAc-GlcA-GalNAc | [M-H]- | 757.2156 | 757.2120 | 6.12 |
| GlcA-GalNAc-GlcA-GalNAc-GlcA-GalNAc | [M-H]- | 1154.3377 | 1154.3315 | 9.17 |
| ∆GlcA-GalNAc-GlcA-GalNAc-GlcA-GalNAc | [M-H]- | 1136.3271 | 1136.3210 | 7.98 |
| ∆GlcA-GalNAc-GlcA-GalNAc-GlcA-GalNAc | [M-2H]^2-^ | 567.6599 | 567.6570 | 7.99 |
|  |  |  |  |  |
| GlcA-GalN | [M-H]- | 354.1042 | 354.1018 | 5.62 |
| ∆GlcA-GalN | [M-H]- | 336.0936 | 336.0916 | 5.14 |

**Table S2:** Differential scanning fluorimetry melts of BcGDH mutants.

| Protein | Melting temperature ^o^C (± SD of triplicates) |
| --- | --- |
| Wild type | 50.8 (± 0.1) |
| E74A | 49.9 (± 0.2) |
| R78A | 48.3 (± 0.1) |
| N176A | 47.8 (± 0.7) |
| N177A | 52.5 (± 0.1) |
| W178A | 53.6 (± 0.6) |
| F181A | 47.9 (± 0.1) |
| Y229A | 50.0 (± 0.1) |
| R285A | 49.5 (± 0.1) |
| Y289A | 48.9 (± 0.3) |
| Y354A | 50.1 (± 0.2) |

**Table S3:** Oligonucleotide primer sequences used for gene amplification

| **Construct** | **SEQUENCE (5’-3’)** |
| --- | --- |
| **PL35_H387A_FWD** | GCGAACCGCGTCAAGCGGCCCAGCATTTGG |
| **PL35_H387A_REV** | CCGTCCAAATGCTGGGCCGCTTGACGCGG |
| **PL35_W419A_FWD** | GCCGATGATTACGCTTTGCCGGGTTTTGCCGATGGGCGCC |
| **PL35_W419A_REV** | CGTCGGGGCGCCCATCGGCAAAACCCGGC |
| **PL35_R076A_Twist_FWD** | GATAGAAACCTTTGCTGTCCATACATATTGCTAGCCATATG |
| **PL35_R076A_Twist_REV** | CCAACGAAGCATTGCTGTGGGTTTG |
| **PL35_E228A_Y232A_Twist_FWD** | CCTCTTTCGGGTAATCCTCGGCAATGCCGAGCG |
| **PL35_E228A_Y232A_Twist_REV** | CGGATGCTCAAAACAGCCCGGCGTTCTTCTTGTTCAG |
| **PL35_Y419A_Twist_FWD** | CTTGACGCGGTTCGCCGCCCTTTGCAATAAG |
| **PL35_Y419A_Twist_REV** | CCCTGTATAAAGACCAGGCGTCCAGCGTTTTTCGTACC |
| **GH154_E074A_FWD** | AAGGACATGACGCATCTGGCAGCCGTCGGTCGC |
| **GH154_E074A_REV** | CAACGTGCGACCGACGGCTGCCAGATGCGTCAT |
| **GH154_R078A_FWD** | GAGGCCGTCGGTGCTACGTTGGCTG |
| **GH154_R078A_REV** | GACGCCAGCCAACGTAGCACCGACG |
| **GH154_N176A_FWD** | GATCGTACCGGCGCATACGCCAACTGGCTG |
| **GH154_N176A__REV** | GCCGGTAAATAACAGCCAGTTGGCGTATGCGCC |
| **GH154_N177A_FWD** | ACCGGCGCATACAACGCCTGGCTGTTATTTAC |
| **GH154_N177A _REV** | CAGGCCGGTAAATAACAGCCAGGCGTTGTATGC |
| **GH154_W178A_FWD** | GGCGCATACAACAACGCACTGTTATTTACC |
| **GH154_W178A _REV** | TCAGGCCGGTAAATAACAGTGCGTTGTTGTATG |
| **GH154_F181A_FWD** | AACAACTGGCTGTTAGCTACCGGCCTG |
| **GH154_F181A _REV** | CGACTCGGTCAGGCCGGTAGCTAACAG |
| **GH154_Y229A_FWD** | TTCAGCATGGATAATGCTAACGCGTAC |
| **GH154_Y229A _REV** | CATCACGTACGCGTTAGCATTATCCATG |
| **GH154_R285A_FWD** | ACGTACCCGGCGTTTGGTGCTTCCGTGACC |
| **GH154_R285A _REV** | ACGATAGGTCACGGAAGCACCAAACGCCGG |
| **GH154_Y289A_FWD** | GGTCGTTCCGTGACCGCTCGTACTGCTG |
| **GH154_Y289A _REV** | GAAAGCAGCAGTACGAGCGGTCACGGAAC |
| **GH154_Y354A_FWD** | GAGTGCGCTGACGGCGCTACTTCGACC |
| **GH154_Y354A _REV** | GCTCCCGGTCGAAGTAGCGCCGTCAGC |

**Table S4:** List of BcPL35 Twist fragments used for mutagenesis.

| **Name** | **SEQUENCE (5’--->3’)** |
| --- | --- |
| R076A | AATATGTATGGACAGCAAAGGTTTCTATCAAACCACCCGAGACTGCTGTTTACCGGTGCTGAGGAAGCGGCGGTCAAACAATTAATCCAGAACAACCGCCTGGCGGGCGAGCTGGCGGAGTTCCTGAAGGCGAAGGCCGACACCCTGGTCATTACCCCGCAAAAACCGTATCTTAAAGACAAGTACGGCAATATTCTGTGGACGAGC**GCC**TCGTATGTTAATCGCCTGGGTACCTTGGCCCTGGCCTACCGGTTGTACGGTGAGCGTAAATACCTGGACGCTGCCAACGAAGCATTGCTG |
| E228A | CATTGCCGAGGATTACCCGAAAGAGGCTGCGGTTATCCTGGACAACGCTGCGAAGTACATGCCGAATTGTCTGAAGCACTTTGCACCGGATGGTGTTTGTTATG**CC**GGTCCGGCGTACTGGGGTTATACCACATCGTACTTGACCCTGTACTTAAAGGCGGTGGCCGATAACGACAACGGTAAGGGCGGGATCGCCCAGTTACCGGGTCTGGAGCGTACTGCGCTCTACCAGAAGCGCACCCTGACTCCGAGCGGTCGCTTATTCAACTTTGGTAACGCAGGCGCGGATGCTCAAAACAG |
| Y232A | CATTGCCGAGGATTACCCGAAAGAGGCTGCGGTTATCCTGGACAACGCTGCGAAGTACATGCCGAATTGTCTGAAGCACTTTGCACCGGATGGTGTTTGTTATGAGGGTCCGGCG**GC**CTGGGGTTATACCACATCGTACTTGACCCTGTACTTAAAGGCGGTGGCCGATAACGACAACGGTAAGGGCGGGATCGCCCAGTTACCGGGTCTGGAGCGTACTGCGCTCTACCAGAAGCGCACCCTGACTCCGAGCGGTCGCTTATTCAACTTTGGTAACGCAGGCGCGGATGCTCAAAACAG |
| Y413A | GCGGCGAACCGCGTCAAGCGCACCAGCATTTGGACGGCGGCACCTTCATCGTGGAAAGCAATGGCGTTTGCTGGACCGAAGATCTGGGTGCCGATGATGCCGCTTTGCCGGGTTTTTGGGATGGGCGCCCCGACGGCCAGCGTTGGAAATATTTCCGCAACAATAACTTTAGCCATAACACGTTGTCCATCGATCATAAGATCCAATACGCGAATGGCGAGGCGTTTGTGTGCGAGGAACACACCGATGCGAAGCAACCGAGCGTTAAGCTGGACATGACCACCCTGTATAAAGACCAGG |

**Table S5**: Gradient conditions for separation of chondroitin and chondrosine enzyme products.

| **Time (min)** | **A (%)** 10 mM ammonium formate  50 mM formic acid  80% acetonitrile  20% water | **B (%)**  10 mM ammonium formate  50 mM formic acid  20% acetonitrile  80% water |
| --- | --- | --- |
| 0 | 100 | 0 |
| 1 | 70 | 30 |
| 10 | 62.5 | 37.5 |
| 10.1 | 0 | 100 |
| 12 | 0 | 100 |
| 12.1 | 100 | 0 |
| 22 | 100 | 0 |

**Table S6**: Parameters for ESI-MSn on the Orbitrap Fusion Tribrid.

| **Parameter (units)** | **Value** |
| --- | --- |
| *ESI* |  |
| Spray voltage: negative ion (V) | 2500 |
| Sheath Gas (Arb) | 45 |
| Aux Gas (Arb) | 10 |
| Sweep Gas (Arb) | 1 |
| Ion Transfer Tube Temp (°C) | 325 |
| Vaporizer Temp (°C) | 250 |
|  |  |
| *MS* |  |
| Detector Type | Orbitrap |
| Orbitrap Resolution | 120K |
| Mass Range | Normal |
| Scan Range (m/z) | 150-2000 |
| RF Lens (%) | 50 |
|  |  |
| *Data-dependent MS2* |  |
| Collision Energy Type | Normalized |
| Isolation Mode | Quadrupole |
| Activation Type | HCD |
| Collision Energy Mode | Stepped |
| Collision Energies (%) | 15,30,45,60,80 |
| Detector Type | Orbitrap |
| Orbitrap Resolution | 30K |

**Table S7:** X-ray data collection and structure statistics

|  | BcPL35 | BcGDH | BcGDHR285A |
| --- | --- | --- | --- |
|  |  |  | + chondrosine |
| ***Data Collection*** |  |  |  |
| Wavelength | 1.541 | 1.541 | 1.541 |
| Space Group | P2_1_2_1_2_1_ | P2_1_ | P2_1_2_1_2_1_ |
| Cell Dimensions |  |  |  |
| *a, b, c* (Å) | 69.284, 84.043, 112.870 | 53.535, 161.37, 88.61 (β=95.70) | 99.55, 105.86, 179.93 |
| Resolution (Å) | 30.00-1.75 (1.78-1.75) | 20.00-2.20 (2.24-2.20) | 20.00-2.60 (2.65-2.60) |
| R_meas_ | 0.083 (0.649) | 0.083 (0.325) | 0.191 (0.994) |
| R_pim_ | 0.031 (0.357) | 0.046 (0.199) | 0.092 (0.500) |
| CC1/2 | 0.998 (0.924) | 0.968 (0.997) | 0.995 (0.844) |
| <I/σI> | 20.6 (1.8) | 13.2 (3.0) | 7.5 (1.3) |
| Completeness (%) | 99.8 (99.5) | 94.1 (80.7) | 98.7 (98.9) |
| Redundancy | 4.9 (3.0) | 2.7 (2.0) | 4.0 (3.9) |
| No. of Reflections | 329,566 | 191,567 | 231,408 |
| No. Unique | 67,073 | 71,486 | 58,307 |
|  |  |  |  |
| ***Refinement*** |  |  |  |
| Resolution (Å) | 1.75 | 2.20 | 2.60 |
| R_work_/R_free_ | 0.185/0.217 | 0.164/0.216 | 0.22/0.26 |
| No. of Atoms | 5,311 | 13,277 | 12,912 |
| Protein | 4,631 | 12,270 | 12,377 |
| Ligand | 2 | n/a | 96 |
| Water | 678 | 956 | 412 |
| *B*-factors | 21.88 | 32.04 | 46.78 |
| Protein | 20.68 | 31.79 | 46.58 |
| Ligand | 16.63 | n/a | 67.28 |
| Water | 30.08 | 35.18 | 43.31 |
| r.m.s.d. |  |  |  |
| Bond Lengths (Å) | 0.007 | 0.006 | 0.002 |
| Bond Angles (°) | 0.929 | 0.804 | 0.583 |
| Ramachandran (%) |  |  |  |
| Preferred | 97.8 | 97.52 | 97.0 |
| Allowed | 2.24 | 2.48 | 2.9 |
| Disallowed | 0.0 | 0.0 | 0.0 |
| PDB ID | 9O3Q | 9O4U | 9NWF |

**Figure S1.** Screening BcPL35 activity. A) Tracking formation of unsaturated products by UV absorbance. CH refers to cCH as was included as a positive control. B) As in panel A but tested two forms of hyaluronic acid. C) FACE analysis of HA degradation products by BcPL35 (PL35) and the *Streptococcus pnuemoniae* hyaluronate lyase (PL8) as a positive control. D) Activity of sulfatases on pNP-sulfate.

**Figure S2**. Full FACE gels from figure 1C and 1D of the main text.

**Figure S3.** BcPL35 kinetics. A) Kinetic analysis of BcPL35 on cCH showing individual replicates. The solid line shows the best-fit line to the Michaelis-Menton model. B) Kinetic analysis of BcPL35 W419A (solid circles) and Y232A (open circles) on cCH showing individual replicates. The solid line shows the best-fit line from linear regression.

**Figure S4**. Enzyme reaction products from cCH digested with BcPL35 analyzed by LC-ESI-MS. A) Extracted ion chromatograms are shown for ions of interest with ion counts scaled relative to the most intense peak for all chromatograms. Chromatograms are for extracted ions in the enzyme reaction (dark colours) or control/no enzyme reactions (light colours). B-D) ESI-MS/MS with HCD was performed in order to identify the extracted ion for the primary catalytic products in each.

**Figure S5**. Activity of BcGH88. A) Activity of BcGH88 on CSD0S. B) Oxidation of NADH by KduD and KduI activity on the 5-keto-4-deoxyuronate produced by BcGH88 from CSΔ0S. C) Kinetics of BcGDH showing individual replicates. Solid lines show best fits from linear regression.

**Figure S6**. Activity of BcGDH on glycosides. A) screen of BcGDH purified from E. coli BL21 (grey bars) or Tuner (black bars) on aryl-glycosides. Error bars represent the standard deviation of triplicate samples. B) Glucuronic acid detection assay for activity on chondrosine.

**Figure S7**. Kinetics of β-glucuronate dehydrogenases. A) Kinetics of BcGDH on 1-*O*-methyl-β-D-glucuronate. Error bars represent the standard deviation of triplicate samples. B) Kinetics of BcGDH on chondrosine showing individual replicates. C) Kinetics of BT_3677 on 1-*O*-methyl-β-D-glucuronate. Error bars represent the standard deviation of triplicate samples. D) Individual replicates of data in panel B. The solid lines in all panels represent the best fit lines by linear regression; dashed lines indicate 95% confidence limits.

**Figure S8**. Structural comparisons of BcGHD. A) The BcGDH tetramer (orange) overlaid with the BD-β-Gal tetramer (grey; PDB ID 8OI4). B) The conserved active site of BcGDH with BD-β-Gal with important residues shown as sticks. C) The BcGDH monomer (orange) overlaid with an AlphaFold model of BT3677 (blue). Important active site residues are shown as sticks.
